# Metabolism of Androstenone, 17β-Estradiol and Dihydrotestosterone in Primary Cultured Pig Hepatocytes and the Role of 3β-Hydroxysteroid Dehydrogenase in This Process

**DOI:** 10.1371/journal.pone.0113194

**Published:** 2015-01-15

**Authors:** Gang Chen, Ying Bai, Li Ren, Dan Zhu, Yanhua Li, Meiying Fang, Huda Al-Kateb, Olena Doran

**Affiliations:** 1 Key Laboratory of Agro-product Quality and Safety, Institute of Quality Standards & Testing Technology for Agro-Products, Chinese Academy of Agricultural Sciences (CAAS), Beijing, China; 2 College of Animal Science and Technology, China Agricultural University, Beijing, China; 3 Department of Pathology, Air Force General Hospital, Beijing, China; 4 Centre for Research in Biosciences, Faculty of Health and Applied Sciences, University of the West of England, Bristol, United Kingdom; Faculty of Biology, Spain

## Abstract

Steroids metabolism plays an important role in mammals and contributes to quality of pig meat. The main objective of this study was to identify metabolites of androstenone, 17β-estradiol and dihydrotestosterone using primary cultured pig hepatocytes as a model system. The role of 3β-hydroxysteroid dehydrogenase (3βHSD) in regulation of steroid metabolism was also validated using trilostane, a specific 3βHSD inhibitor. Steroid glucuronide conjugated metabolites were detected by liquid chromatography time of flight mass spectrometry (LC-TOF-MS). 3βHSD enzyme was essential for metabolism of androstenone to 5α-androst-16-en-3β-ol, which then formed androstenone glucuronide conjugate. Metabolism of 17β-estradiol was accompanied by formation of glucuronide-conjugated estrone and glucuronide-conjugated estradiol. The ratio of the two metabolites was around 5∶1. 3βHSD enzyme was not involved in 17β-estradiol metabolism. 5α-Dihydrotestosterone-17β-glucuronide was identified as a dihydrotestosterone metabolite, and this metabolism was related to 3βHSD enzyme activity as demonstrated by inhibition study.

## Introduction

Steroid hormones are essential for a range of physiological processes in mammals. They act as body chemical messengers, and play critical role in organism development and maturation [1]. Steroids also play an important role in regulation of pig metabolism and pig meat quality. Androgen metabolism results in production of a group of non-hormonal androstanes, which act as pheromones and influence physiology or behavior of mammals [4]. Accumulation of one of the androstanes, 5α-androst-16-ene-3-one (androstenone) in adipose tissue of entire male pigs is associated with a pork quality defect, boar taint, which is unpleasant ‘urine-like’ odour [5]. Other compounds contributing to boar taint are skatole and indole which are formed during tryptophan metabolism [2]. Boar taint can be prevented by surgical castration which reduces the level of steroids, including androstenone [6]. However, due to the EU initiative to ban surgical castration by 2018, boar taint becomes an increasing issue for international pig meat industry and introduces the challenge of reduction of androstenone level by the means other than castrations [7]. Decreasing androstenone level in pork can be achieved either via reduction of the rate of androstenone biosynthesis, or via enhancing the rate of androstenone metabolism [8]. It has been reported that enzymes of hydroxysteroid dehydrogenase family (HSDs), namely 3βHSDs and 17βHSDs, play the central role in steroid metabolism [9]. Data of the literature showed that porcine 3βHSD enzyme catalyzes the conversion of androstenone to its hydroxyl form in pig liver [10]. Testosterone is the main active androgen. It can be either irreversibly converted to estrogens by aromatase, or can be transformed to other active form such as dihydrotestosterone [9]. Testosterone metabolism in pig hepatocytes has been studied previously and it was established that 4-androstene-3,17-dione was its main metabolite [13]. 17β-Estradiol and dihydrotestosterone are also known as bioactive steroids of the endocrine systems. 17β-Estradiol directly influences reproduction of sows and indirectly influences off-springs development in terms of body weight and composition [3]. Biological role of dihydrotestosterone in pigs has not been extensively studied and has not been fully understood. To the best of our knowledge, relationship between 3βHSD and 17β-estradiol and dihydrotestosterone metabolism has not been investigated.

One of the issues with investigation of steroids metabolism is a lack of appropriate analytical methods which would allow to differentiate steroids from their metabolites. Physiological concentrations of steroid metabolites are very low and their structure is very similar to the structures of corresponding steroids [10], [11]. During the last decade, liquid chromatography mass spectrometry with electrospray ionization (ESI) has been receiving increasing attention in relation to its application to steroids profile analysis [12]. Mass spectrometry with the time of flight mass analyzer (TOF) is another promising technology for identification of steroid metabolites because of its high resolution, high accuracy in mass detection, and a wide range of m/z scan.

Aims of this study were to use time of flight mass spectrometry coupled with liquid chromatography (LC-TOF-MS) in order to (i) characterize products of androstenone, 17β-estradiol and dihydrotestosterone metabolism, and (ii) investigate the role of 3βHSD enzyme in metabolism of androstenone, 17β-estradiol and dihydrotestosterone using primary cultured pig hepatocytes as a model system.

## Materials and Methods

### Chemicals and reagents

Androstenone, 17β-estradiol, dihydrotestosterone, β-estradiol 17-(β-D-glucuronide) sodium salt, β-glucuronidase from bovine liver (type B-1), 3α-androstenol, 3β-androstenol, apigenin, and trilostane were from Sigma-Aldrich (Shanghai, China). Stock solutions of the steroids were prepared in methanol at concentrations of 1 g/L. Working solutions of steroids were prepared by diluting the stock solutions in methanol. All other reagents and solvents (HPLC grade) were purchased from Fisher Scientific (service in Beijing, China). Cell culture media and other cell culture reagents were purchased from Hyclone (Beijing, China).

### Isolation of primary hepatocytes and treatments with steroids

Primary pig hepatocytes were isolated from Large White male pigs (3–5 days old) using procedure described by Chen et al. [13]. The experimental protocol for using animals was approved by the Animal Ethics Committee of the China Agricultural University, Beijing, China (permission number: 2011-11-23-1). Cell viability was assessed by 0.2% trypan blue exclusion and was greater than 90% in all the cases. Approximately 5×10^6^ cells were plated into 10 cm Petri dishes with 10 ml of Dulbecco modified Eagle’s medium (DMEM) containing 20% fetal bovine serum (FBS) supplemented with insulin (10 mg/L), penicillin (100 U/mL) and streptomycin (100 µg/mL). Hepatocytes were incubated for 24 h in a humidified atmosphere at 37°C with 5% of CO_2_.

After the incubation, the primary pig hepatocytes were washed three times with phosphate buffer saline solution (PBS) before they were treated with steroids and/or inhibitors. In steroid metabolism studies, hepatocytes were incubated in 10 ml of basal growth medium containing phenol red-free DMEM with 10% of charcoal treated FBS fortified with 10 µM (final concentration) of androstenone, 17β-estradiol or dihydrotestosterone. In the inhibition experiments, 10 µM (final concentration) of trilostane or apigenin, the specific inhibitors of 3βHSD and 17βHSD respectively, were added to hepatocytes simultaneously with androstenone, 17β-estradiol or dihydrotestosterone. Solutions of both, the inhibitors and steroids, were prepared in methanol. Concentration of methanol in the cell culture medium was always less than 1%, and it did not have any effect on hepatocyte metabolism. The concentration choice was based on results of cytotoxicity test. In the cytotoxicity text, the steroids were incubated with different concentrations of inhibitors (1, 10, 25 and 50 µM) for 48 h. The cell viability was analyzed using CellTiter-Glo Luminescent Cell Viability Assay (Promega, Madison, WI) reagent following manufacture’s protocol. The cell culture medium was collected at the following time points: 0, 6, 12, 18, 24, 30, 36, 42 and 48 h after adding steroids and/or inhibitors, and was stored at −80°C until metabolites analysis. Cells treated with basal growth medium were used as control. The experiments were repeated in three different days. Experiments on each of the three days were conducted in triplicate.

### Steroid metabolites extraction

Extraction procedure for steroid metabolites was as follows: two mL of cell culture medium was taken in duplicate from each hepatocytes incubation and split into two samples, 1 mL per sample. One sample was treated with 0.1 mL of 30% trichloroacetic acid (TCA) to precipitate protein. The protein precipitation procedure was optimized by analyzing protein residues using HPLC at ultraviolet wavelength (240 nm). Another sample was pre-treated with β-glucuronidase prior to protein precipitation. The pre-treatment involved incubation with 2 mL of PBS buffer (pH = 5.0) in the presence of β-glucuronidase (2.08 µkat) for 16 h at 37°C. The samples were centrifuged at 17226 g for 10 min. All of the supernatants were collected and loaded onto a SPE cartridge (Bond Elut-C18, 500 mg/3 mL, Agilent, Lake Forest, CA, USA) preconditioned with 3 mL of methanol and water. The cartridges were washed with 3 mL of water and 3 mL 5% of methanol and then vacuum dried. The analytes were eluted with 9 mL of acetonitrile (ACN). The elutants were evaporated under nitrogen and reconstituted with 400 µL of 62.5% methanol. Solvents were passed through 0.22 µm filter paper (Jinteng company, Tianjin, China), and then transferred to inserts for analysis. The recoveries were determined by analyzing spiked samples in a basal medium. The recoveries for androstenone, 17β-estradiol and dihydrotestosterone were in a range of 60–95%.

### Mass spectrometry analysis

Steroid metabolites were analyzed by LC-TOF-MS (QSTAR Elite, AB Sciex, ON, Canada) equipped with an electrospray ion source (TurboIonSpray) and controlled by Analyst QS 2.0 software. ESI source was run on both, a positive and negative mode. Spectrometry parameters were as follows: ion source GS1 and GS2 were 0.41 and 0.34 MPa, respectively. Curtain gas was 0.14 MPa. Collision gas was 0.04 MPa. All the gases were supplied with nitrogen (purity≥99.995%). IonSpray voltage was 5500 V (ESI+) and −4200 V (ESI–). Ion source temperature was 500°C. Declustering Potential (DP) was 60 V (ESI+) and −60 V (ESI–). The Q1 mass monitoring was conducted using a scan mode in the range from 200 to 600 m/z. Mass intensity above 100 counts was set for acquiring product ions on TOF analyzer. An information dependent acquisition (IDA) method was set to optimize data acquisition.

Chromatographic separation was conducted using HPLC coupled to mass spectrometry (1200 SL, Agilent Technologies, USA) equipped with a binary pump, vacuum solvent degasser, column oven and autosampler (Agilent Technologies, Waldbronn, Germany). The column used was Zorbax Eclipse Plus C18 (2.1×100 mm, 3.5 µm) with a guard column (2.1×12.5 mm, 5 µm) (Agilent Technologies, USA). For the ESI+ mode, the mobile phase was 0.1% formic acid in water (A) and methanol (B). For the ESI– mode, the mobile phase was 0.1% ammonium in water (A) and methanol (B). The linear gradient used was as follows: 50% to 95% solvent B at 0–5 min; 95% solvent at 9 min, 50% solvent B at 9.1 and 15 min. The flow rate was 300 µL/min, and the sample injection volume was 20 µL.

### Statistical analysis

Data were analyzed by the Statistical Analysis System, version 9.0 (SAS Institute, Cary, NC, USA). Anova procedure was used to evaluate the group differences, and the means of groups were compared by Duncan’s multiple comparison. P<0.05 was regarded as statistically significant.

## Results

### Cytotoxicity study

The results of cell viability incubated at different concentration of steroids and HSD inhibitors were given in Table 1. The cell survival rate of 80% was regarded as a threshold for evaluating the chemicals with or without toxicological effect. It can be seen that steroids and inhibitors at 10 µM have cell viability higher than 80%, and therefore was chosen for further experiment.

**Table 1:** Cytotoxicity of steroids and HSD inhibitors in isolated pig hepatocytes.

Concentration (µM)	Androstenone	17β-Estradiol	Dihydrotestosterone	Trilostane	Apigenin
1	90.6±3.8	109.2±4.8	96.7±0.1	91.8±0.1	97.7±3.2
10	89.0±7.2	91.6±0.4	84.3±3.9	82.4±2.1	83.5±5.9
25	77.2±0.6	89.8±8.0	79.1±2.2	79.1±2.9	64.2±1.7
50	78.7±6.8	83.6±1.6	76.1±4.7	69.2±1.8	69.8±0.9

Cells (≈5×10^6^ cells/plate) were incubated in the presence of different concentrations of steroids and inhibitors (0, 1, 10, 25 and 50 µM) for 48 h. Cell viability was evaluated by analyzing the luminescence intensity (RLU) normalized to mean value of cells without treatment. Data presented as Mean ± SD. Effects of steroids and inhibitors at different concentrations vs. cell viability were evaluated by one-way anova. Cell viability did not differ significantly (p>0.05) in the presence of steroids and inhibitors at any of the concentrations studied. The experiments were conducted in three independent batches, with triplicate repeats for each experiment.

### Androstenone metabolism

**Figure 1:**
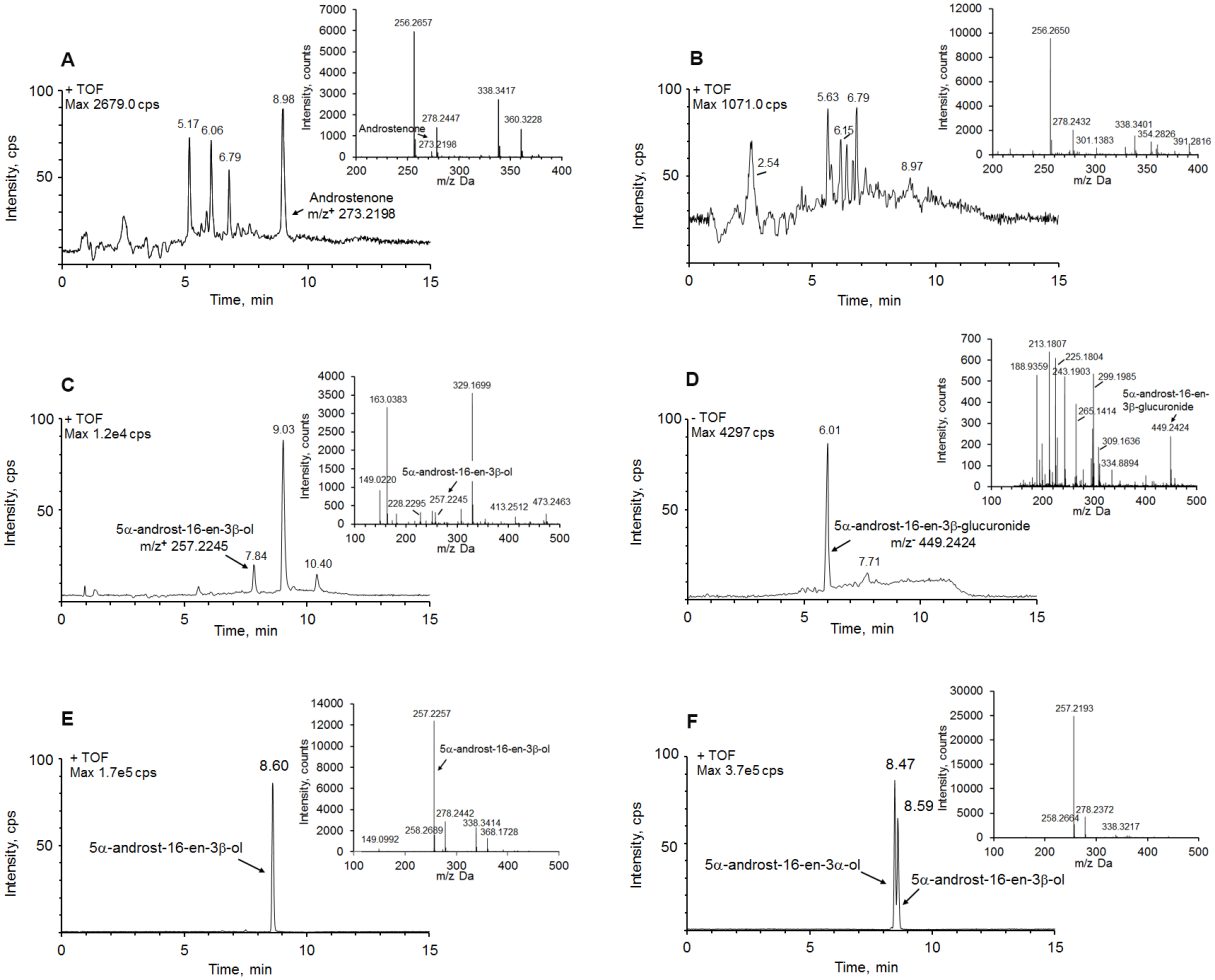
Ion chromatograms and mass spectra (inset) of androstenone and its metabolites. (**A**) androstenone in the medium in absence of isolated hepatocytes; (**B**) androstenone in the medium in the presence of isolated hepatocytes. No androstenone was found after 24 h of cell culture; (**C**) identified androstenone metabolite (m/z+ = 257.2245). The samples were analyzed in ESI+ ionization mode; (**D**) identified androstenone metabolite 5α-androst-16-en-3β-glucuronide (m/z− = 449.2424) in ESI– ionization mode; (**E**) identified androstenone metabolite 5α-androst-16-en-3β-ol after enzyme hydrolysis by β-glucuronidase; (**F**) a mixture of authentic standards 5α-androst-16-en-3α-ol (3α-A) and 5α-androst-16-en-3β-ol (3β-A).

Figure 1A presents data on abundance of androstenone precursor ion (m/z+ = 273.2198) in the incubation medium before cell culturing. A time-course of androstenone metabolism showed that androstenone was non-detectable in the cell culture medium after 24 h of incubation (Figure 1B). LC-TOF-MS analysis of androstenone metabolites in ESI+ ionization mode identified a metabolite ion (m/z+ = 257.2245), which corresponds to a precursor ion of androstenone without a 3-keto group (Figure 1C). Analysis of androstenone metabolite in ESI– ionization mode demonstrated the presence of another ion with m/z− = 449.2424 (Figure 1D). A molecular formula of this ion was determined using MetID software. The analysis demonstrated that this ion corresponds to a glucuronide-conjugated androstenone (A–G). Further investigation was conducted to determine association between the results obtained with the two different ionization approaches. The fraction of m/z+ = 257.2245 from post-HPLC column was collected and analyzed in ESI– mode. The results demonstrated a sole mass spectrometric peak of m/z− = 449.2424, with the retention time corresponding to the peak of androstenone metabolite in the cultured medium samples analyzed in ESI– mode. These experiments confirmed that the ion m/z+ = 257.2245 is the glucuronide-conjugated androstenone which lost glucuronide structure (C_6_H_8_O_6_, MW = 176) in ESI+ ionization.

It is known that glucuronidation occurs at the hydroxyl group of compounds. Since androstenone does not have a hydroxyl group, we suggested that androstenone was hydroxylated by a specific enzyme at the 3-keto group first, and then was subjected to glucuronidation. To check this hypothesis, β-glucuronidase was added to the cell culture medium. Presence of hydroxylated androstenone with the m/z+ = 257.2257 was determined in the medium after incubation with β-glucuronidase using LC-TOF-MS (Figure 1E). Comparing the samples with authentic standards of 5α-androst-16-en-3α-ol (3α-A) and 5α-androst-16-en-3β-ol (3β-A) allowed to establish the presence of hydroxylated androstenone in the medium after incubation with β-glucuronidase. Therefore, this hydroxylated androstenone was defined as 3β-A (Figure 1F). These experiments confirmed that androstenone was hydroxylated to 3β-A prior to glucuronidation reaction.

The inhibitory study demonstrated that incubation of the primary isolated hepatocytes in the presence of 10 µM of trilostane, the specific inhibitor of 3βHSD, resulted in 70% decrease in the formation of androstenone metabolite in the cell culture medium. Incubation of hepatocytes with 10 µM of apigenin, the specific inhibitor of 17βHSD, did not affect significantly the level of androstenone metabolite (Table 2).

**Table 2:** Peak heights (cps) of the ion chromatograms of steroids metabolites in cell culture medium.

Metabolites	C	Apigenin	Trilostane
Androstenone glucuronide	3.4±0.5	3.7±0.9	1.0±0.6**
Estrone glucuronide	16.5±2.2	15.3±1.1	13.5±1.7
Estradiol 3β-glucuronide	3.1±0.8	2.6±0.1	2.7±0.4
Dihydrotestosterone 17β-glucuronide	11.8±1.1	11.5±0.5	1.6±0.2**

Data are presented as Mean ± SD. (**) presents statistical significance of differences between groups at p<0.01. The peak height values are presented after division by 1000 (cps/1000).

C = steroid metabolites analyzed after 48 h incubation of cell culture in absence of enzyme inhibitors. Apigenin = steroid metabolites analyzed after 48 h of cell culture in the presence of apigenin, the specific inhibitor of 17βHSD. Trilostane = steroid metabolites analyzed after 48 h of incubation of cell culture in the presence of trilostane, the specific inhibitor of 3βHSD. The cell density was approx. 5×10^6^ cells/plate. The experiments were conducted in three independent batches with triplicate repeats for each experiment.

### 17β-Estradiol metabolism

**Figure 2:**
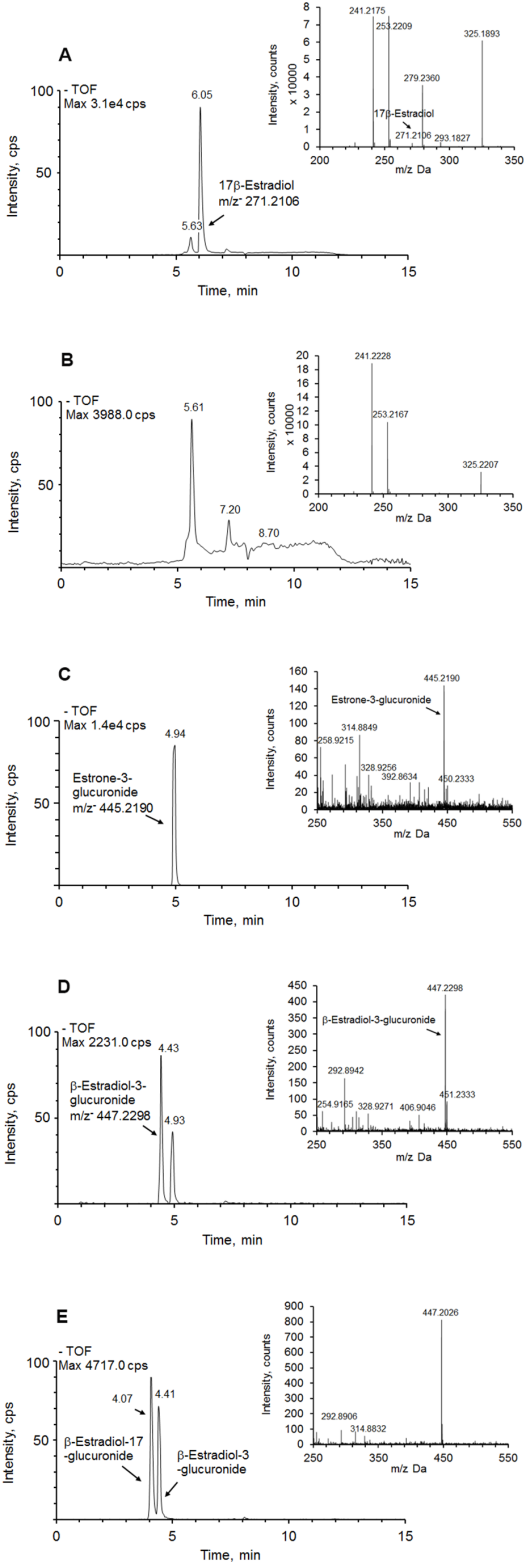
Ion chromatograms and mass spectra (inset) of 17β-estradiol and its metabolites. (**A**) 17β-estradiol in the medium in absence of isolated hepatocytes; (**B**) 17β-estradiol in the medium in the presence of isolated hepatocytes. No 17β-estradiol was found after 6 h of cell culture; (**C**) identified 17β-estradiol metabolite estrone-3-glucuronide (m/z− = 445.2190). The samples were analyzed in ESI– ionization mode; (**D**) identified 17β-estradiol metabolite β-estradiol-3-glucuronide (m/z− = 447.2298) in ESI– ionization mode; (**E**) a mixture of authentic standards β-estradiol-17-glucuronide and β-estradiol-3-glucuronide.

Abundance of 17β-estradiol precursor ion (m/z− = 271.2106) in the medium before cell culturing is shown in Figure 2A. 17β-Estradiol was completely metabolized by pig hepatocytes after 6 h of incubation in the medium (Figure 2B). Investigation of 17β-estradiol metabolites by LC-TOF-MS established the presence of two compounds with m/z− 445.2190 and 447.2298, which correspond to molecular ions of glucuronide-conjugated estrone (E1-G) (Figure 2C) and glucuronide-conjugated estradiol (E2-G) (Figure 2D) respectively. The ratio of E1-G to E2-G was 5∶1. When the cell culture medium was hydrolyzed by β-glucuronidase, both estrone and 17β-estradiol were detected. The ratio estrone: 17β-estradiol was similar to the ratio E1-G: E2-G. Using β-estradiol-3′-glucuronide and β-estradiol-17′-glucuronide authentic standards, this study established that glucuronidation of E2-G occurred at 3′-hydroxyl group (Figure 2E).

There was no significant inhibition of E2-G and E1-G formation in the presence of trilostane and apigenin (Table 2).

### Dihydrotestosterone metabolism

**Figure 3:**
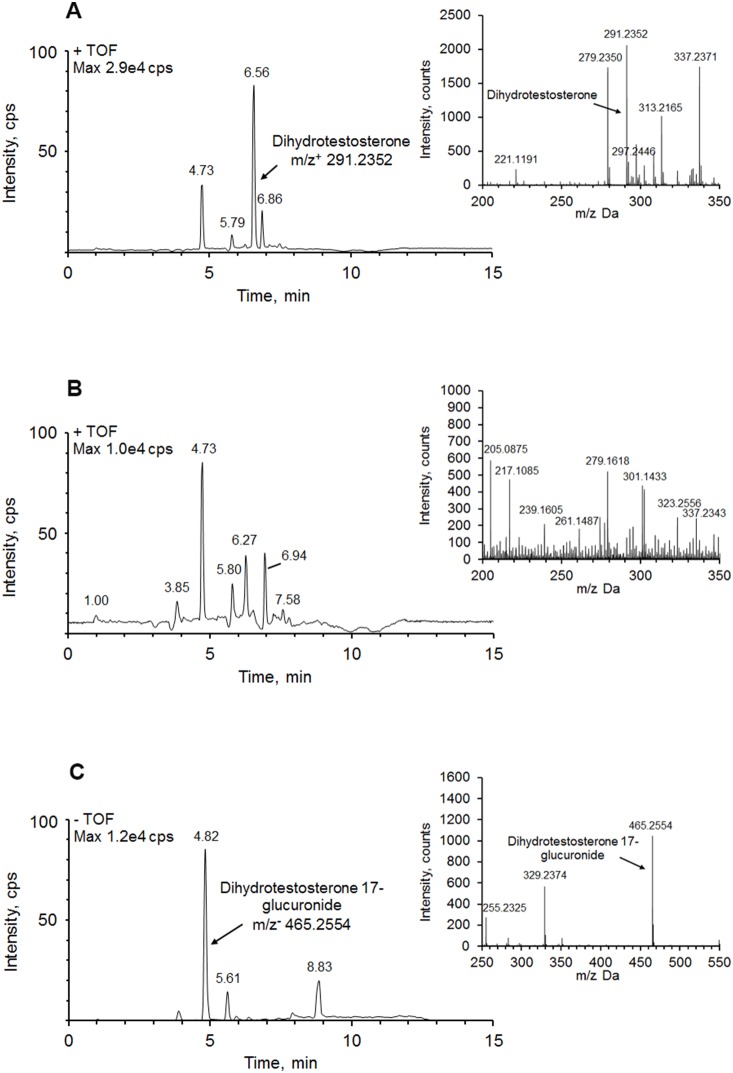
Ion chromatograms and mass spectra (inset) of dihydrotestosterone and its metabolite. (**A**) dihydrotestosterone in the medium in absence of isolated hepatocytes; (**B**) dihydrotestosterone in the medium in the presence of isolated hepatocytes. No dihydrotestosterone was found after 6 h of cell culture; (**C**) identified dihydrotestosterone metabolite dihydrotestosterone-17-glucuronide (m/z− = 465.2554). The samples were analyzed in ESI– ionization mode.

Abundance of dihydrotestosterone precursor ion (m/z+ = 291.2352) in the medium before cell culturing is presented in Figure 3A. Similarly to 17β-estradiol, dihydrotestosterone was also rapidly metabolized by pig hepatocytes after 6 h of incubation (Figure 3B). LC-TOF-MS analysis identified a metabolite with m/z− = 465.2554 (RT = 4.82 min) in ESI– mode (Figure 3C). Molecular formula calculation allowed to establish that this compound matches glucuronide-conjugated dihydrotestosterone (DHT-G). Since dihydrotestosterone contains only one 17β-hydroxyl group, the metabolite was defined as 5α-dihydrotestosterone-17β-glucuronide. Experiments with hydrolysis of the cell culture medium by β-glucuronidase provided further confirmation of presence of glucuronide structure.

Enzyme inhibition study showed that trilostane reduces DHT-G formation in the hepatocytes by almost 86% (Table 2). Incubation with apigenin did not have any effect on DHT-G level.

## Discussion

The level of steroid hormones in pigs influences animal growth rate and fat accumulation, which has impact on economics of pig production [14]. Moreover, an excessive accumulation of androstenone in pig adipose tissue contributes to an unpleasant odour of meat from some entire male pigs which makes the meat unsuitable for human consumption [5]. Therefore, developing strategies for controlling androstenone level in pork is one of the key challenges of pig industry. Accumulation of androstenone in pig adipose tissue can be due to a high rate of androstenone biosynthesis and/or low rate of androstenone metabolism. Therefore, understanding the pathways controlling metabolism of androstenone in pigs is important for improving meat quality. In addition to androstenone, the other two steroids, 17β-estradiol and dihydrotestosterone can have an impact on pig physiology [15], [16]. It has been recently reported that 17β-estradiol and dihydrotestosterone can alter porcine nuclear receptor expression and thus modulate activity of enzymes involved in steroid metabolism including enzymes controlling androstenone metabolism [17].

The present study used LC-TOF-MS to investigate metabolism of androstenone, 17β-estradiol, and dihydrotestosterone in isolated pig hepatocytes as an *in vitro* model. The results showed that androstenone formed a 3β-androstenol structure during the phase I metabolism (reduction). The inhibition study with trilostane and apigenin as specific inhibitors of 3βHSD and 17βHSD respectively, showed that 3βHSD but not 17βHSD is the key enzyme catalyzing the first stage of the hepatic androstenone metabolism. This finding is consistent with previous reports that β-androstenol is the main product of pig hepatic androstenone metabolisms, and that the process of conversion of androstenone to β-androstenol is catalyzed by 3βHSD [8], [18]. Our study also investigated the second stage of androstenone metabolism (conjugation) and established the formation of glucuronidated androstenone structure. Previous reports suggested the existence of glucuronidated androstenone form on the basis of observation that 3α- and 3β-androstenol glucuronide are present in pig urinary system [1], and the formation of conjugates may facilitate the metabolic clearance of the steroids [19]. The present study provided direct evidence of existence of androstenone glucuronide structure in experiments on isolated pig hepatocytes.

Data of the literature on the other main steroid hormone, testosterone report 4-androstene-3,17-dione as the main product of the stage I of the hepatic testosterone metabolism [13]. However, the phase II of the porcine hepatic testosterone metabolism has not been fully investigated, and the formation of conjugated testosterone metabolite cannot be excluded. Some research group observed the formation of androstenone sulfoconjugate in primary cultured porcine hepatocytes. These data were obtained using an analytical approach for sample hydroxylation and GC-MS derivatization [20]. In the present study, formation of sulfoconjugated androstenone was not observed. One explanation for this discrepancy might be tissue-specific formation of different androstenone conjugates. Sulfoconjugated androstenone (but not 16-androstene glucuronide conjugates) was reported to be present in pig plasma and testis [19]. Another reason for discrepancy between results of this paper and data of the literature might be the fact that *in vitro* cell culture systems have a lower concentration of sulfate when compared to *in vivo* conditions, which does not facilitate sulfo-conjugation. Sinclair et al. (2005) suggested that glucuronidation of 16-androstene steroids is the main route of their metabolic clearance [19]. This hypothesis is supported by finding of this paper that androstenone glucuronide is a predominant hepatic conjugate which carry out the main clearance functions in pigs. Our finding is in a line with other papers which reported the presence of androstenone glucuronide in urine [1]. To summarize, the present paper suggests that androstenone metabolic pathway includes (i) transformation of androstenone to 3-hydroxyl structure by 3βHSD enzyme; (ii) sulfoconjugation of androstenone, which might be important for storage of androstenone in testis, and (iii) metabolism of androstenone 3-hydroxyl structure to glucuronide conjugate in the liver. The glucuronide conjugated androstenone can be excreted with urine.

Although enzymes of HSD family are highly expressed in pig liver [8], [21], the physiological role of pig hepatic HSD has not been fully understood. The present study investigated the role of the two HSD enzymes, 3βHSD and 17βHSD, in metabolism of two steroids, 17β-estradiol and dihydrotestosterone in isolated pig hepatocytes. It was found that over 80% of 17β-estradiol was oxidized at C-17 position to form estrone, with follow-on glucuronidation at 3′-hydroxyl group and formation E1-G. Less than 20% of 17β-estradiol underwent direct phase II metabolism and formed E2-G. The observation that E1-G is the main metabolite in 17β-estradiol metabolism is somewhat surprising, since the reduction of keto-steroid is the main reaction in the hepatic tissue [22]. The enzyme inhibition experiments undertaken in the present study showed that 3βHSD and 17βHSD are not involved in 17β-estradiol metabolism. Regarding the second stage of 17β-estradiol metabolism, the present study demonstrated that glucuronidation of 17β-estradiol occurred at 3-hydroxyl but not at 17-hydroxyl group. A mechanism of this stereo selection remains unknown. It has been reported that glucuronidation of steroids is catalyzed by UDP-glucuronosyltransferase (UGT) [23]. The stereo selection depends on UGT enzyme sub-type and it is tissue-specific. It was previously reported that sulfoconjugated estrone (E1S) was the main metabolite and a storage form of estrogens in pigs [19]. The present study did not detect E1S in the *in vitro* cell culture system. This suggest that sulfation of estradiol may occur in other organs, such as testis, which is known as the main organ of steroids genesis [19].

Dihydrotestosterone is one of the most active androgens in the endocrine system of various species [24]. This study reported the formation of glucuronide conjugated dihydrotestosterone in primary cultured pig hepatocytes. Formation of DHT-G was greatly inhibited by trilostane, the specific 3βHSD inhibitor, whereas apigenin, the inhibitor of 17βHSD, did not have any effect on the DHT-G formation. It was previously reported that dihydrotestosterone can be metabolized to 5α-androstane-3α,17β-diol (3α-Diol) and 5α-androstane-3β,17β-diol (3β-Diol) in a purified enzyme system [22]. These metabolites were not found in the present study. One explanation for discrepancy between data of the literature and this study might be the use of different experimental systems: the processes taking place in purified enzyme systems might be different to the processes taking place in primary cultured hepatocytes. Another possible explanation for this discrepancy might be a sequential metabolic pathway of dihydrotestosterone. For example, 3α-Diol and 3β-Diol metabolites may undergo further glucuronidation and sulfation as reported by Stephan et al. (2004) [22]. It has also been reported that DHT-G can be further metabolized at 3 keto-group to form 3α-androstanediol-17-glucuronide [25]. Considering that DHT-G only accounts for 1/6 of total dihydrotestosterone, formation of other dihydrotestosterone metabolites cannot be excluded. Lack of clarify regarding dihydrotestosterone metabolism suggests its multiple and complex metabolic pathway.

### Conclusions

This study employed an effective time of flight mass spectrometry approach to investigate metabolism of steroids androstenone, 17β-estradiol and dihydrotestosterone in isolated pig hepatocytes as a model system. The study provided novel data on pig hepatic steroid metabolism. In particular, this is the first report which directly confirms the formation of glucuronide-conjugated androstenone and involvement of 3βHSD in this process. The study also reported involvement of dehydrogenase enzymes in regulation of 17β-estradiol and dihydrotestosterone metabolism in pig hepatocytes.
